# Immunofibroblasts regulate LTα3 expression in tertiary lymphoid structures in a pathway dependent on ICOS/ICOSL interaction

**DOI:** 10.1038/s42003-022-03344-6

**Published:** 2022-05-04

**Authors:** Saba Nayar, Elena Pontarini, Joana Campos, Onorina Berardicurti, Charlotte G. Smith, Saba Asam, David H. Gardner, Serena Colafrancesco, Davide Lucchesi, Rachel Coleby, Ming-May Chung, Valentina Iannizzotto, Kelly Hunter, Simon J. Bowman, Gianluca Carlesso, Ronald Herbst, Helen M. McGettrick, Jeff Browning, Christopher D. Buckley, Benjamin A. Fisher, Michele Bombardieri, Francesca Barone

**Affiliations:** 1 Centre for Translational Inflammation Research, Institute of Inflammation and Ageing, College of Medical & Dental Sciences, University of Birmingham Research Laboratories, Queen Elizabeth Hospital, Birmingham, B15 2WB UK; 2 National Institute for Health Research (NIHR) Birmingham Biomedical Research Centre and Department of Rheumatology, University Hospitals Birmingham NHS Foundation Trust, Birmingham, UK; 3 Birmingham Tissue Analytics, Institute of Translational Medicine, University of Birmingham, Birmingham, UK; 4 Centre for Experimental Medicine and Rheumatology, William Harvey Research Institute, Queen Mary University of London, London, EC1M 6BQ UK; 5 Rheumatology Unit, Department of Biotechnological and Applied Clinical Science, University of L’Aquila, L’Aquila, Italy; 6 Rheumatology Unit, University of Rome, Sapienza, Italy; 7 Early Oncology ICA, AstraZeneca, One Medimmune Way, Gaithersburg, MD 20878 MD USA; 8 Departments of Microbiology and Rheumatology, Boston University School of Medicine, Boston, MA USA; 9 Kennedy Institute of Rheumatology, University of Oxford, Oxford, UK; 10 Candel Therapeutics, Needham, Boston, MA USA

**Keywords:** Chronic inflammation, Tumour-necrosis factors

## Abstract

Immunofibroblasts have been described within tertiary lymphoid structures (TLS) that regulate lymphocyte aggregation at sites of chronic inflammation. Here we report, for the first time, an immunoregulatory property of this population, dependent on inducible T-cell co-stimulator ligand and its ligand (ICOS/ICOS-L). During inflammation, immunofibroblasts, alongside other antigen presenting cells, like dendritic cells (DCs), upregulate ICOSL, binding incoming ICOS + T cells and inducing LTα3 production that, in turn, drives the chemokine production required for TLS assembly via TNFRI/II engagement. Pharmacological or genetic blocking of ICOS/ICOS-L interaction results in defective LTα expression, abrogating both lymphoid chemokine production and TLS formation. These data provide evidence of a previously unknown function for ICOSL-ICOS interaction, unveil a novel immunomodulatory function for immunofibroblasts, and reveal a key regulatory function of LTα3, both as biomarker of TLS establishment and as first driver of TLS formation and maintenance in mice and humans.

## Abstract (web summary)

The study suggests a link between ICOS-ICOSL signaling and LTa3 induction on T cells. LTa3 subsequently activates fibroblasts in the salivary gland, leading to formation of tertiary lymphoid tissues (TLS).

## Introduction

Complex aggregates of lymphocytes, termed ectopic or tertiary lymphoid structures (TLS)^1^ are often detected at sites of chronic inflammation. TLS predominantly house activated memory T and B cells, and often develop hyperactive germinal centers (GCs), contributing to local expansion of autoreactive B cell clones and, in some cases, escape of malignant B cell clones and development of lymphoma^1–3^. Fibroblasts that display immuno-modulatory functions or “immunofibroblasts” have been recently recognized as key determinants of TLS establishment and maintenance in the tissue^1,3–7^. This population, defined by the expression of PDGFRα and β, podoplanin (PDPN), FAP-1, ICAM-1 and VCAM-1 is responsible for the production of lymphoid cytokine and chemokines in the context of TLS (RFE).

We and others have previously demonstrated that sustained lymphoid chemokine production by immunofibroblasts, in both SLOs and TLS, is dependent on the engagement of lymphotoxin (LT) and TNFα^8–23^. Within SLOs, this entails the differential contributions of soluble (LTα3) and membrane-bound form (LTα1β2) of LT and their respective receptors, TNFR1/2 and LTβR. Mice deficient for either LTα3, LTα1β2 or LTβR develop spleen architectural defects and broader loss of SLOs. The defects observed in *Ltα* and *Ltβr* knockouts are more severe than those described in the *Ltβ* deficient mice, suggesting that LTα3 signalling through TNFR1/2 cooperates with LTβR signalling to enable spleen and SLO development as well as maintain their organization^13–21,24–35^. In the lymphoid tissue of the nasal mucosa and in the fat associated lymphoid tissue, signals mediated by the TNFR1 seem to be more important than those mediated by LTβR, suggesting different hierarchies for the engagement of these two pathways at distinct anatomic sites^33,36,37^. A correlation between the ectopic expression of LTα3, LTα1β2, lymphoid chemokines and the progressive lymphoid organization, within TLS, has been observed in autoimmune experimental mouse models and human autoimmune diseases^38–47^. Moreover, ectopic expression of TNFα and LTα has been shown to induce TLS formation, supporting a causative role for these cytokines in tertiary lymphoneogenesis and ectopic GC formation^48–51^.

We have recently demonstrated that the establishment of an immunofibroblast network, capable of supporting lymphocyte localization and survival at ectopic sites, precedes TLS formation, following a cytokine program that is dependent, in its earliest stages, on a unique cytokine cascade that includes IL-13 and IL-22^8,52^. Those and other pro-inflammatory cytokines, such as IL-17, are known to act as surrogates for LT in early TLS establishment. Nonetheless, it appears evident that the full maturation of the immunofibroblast network is dependent on the production of LT by incoming lymphocytes^8–12,52^. The differential role of the membrane versus the soluble form of LT during TLS formation has yet to be determined. Moreover, there is no clarity on the signals required to induce and sustain LT production in TLS, once the first contact between immune cells and fibroblast is established.

In this manuscript, using an inducible model of TLS formation in murine salivary glands, that mimics histological features of Sjögren’s syndrome (a disease characterized by the presence of sicca syndrome (dryness of mouth and eyes) and systemic autoimmunity^46^), we established that LTα3 mediated signals precede and overcome those mediated by LTα1β2 in driving TLS. We also demonstrated that immunofibroblasts play a key role in this process, providing inducible T cell co-stimulator ligands (ICOSL) to activate incoming (ICOS+) activated lymphocytes and initiate LTα3 production in the TLS. We confirmed the sequential engagement of this pathway both in vivo in our model of TLS and in vitro, using human salivary glands isolated from patients with Sjogren’s syndrome, demonstrating that, blocking ICOS/ICOSL interaction, once this is engaged in the salivary glands, is still able to suppresses LTα3 production, reversing T cell activation and, in vivo, abrogating TLS formation. Finally, we describe a role for LTα3 expression as biomarker in pSS. Taken together, these data provide evidence for a novel immuno-regulatory role for immunofibroblasts in the context of chronic inflammation, demonstrating a pathogenic link between immunofibroblasts, aberrant T cell costimulation and lymphotoxin production in TLS associated diseases.

## Results

### T cell derived lymphotoxin alpha (LTα3) regulates early chemokine production in TLS

**Fig. 1:**
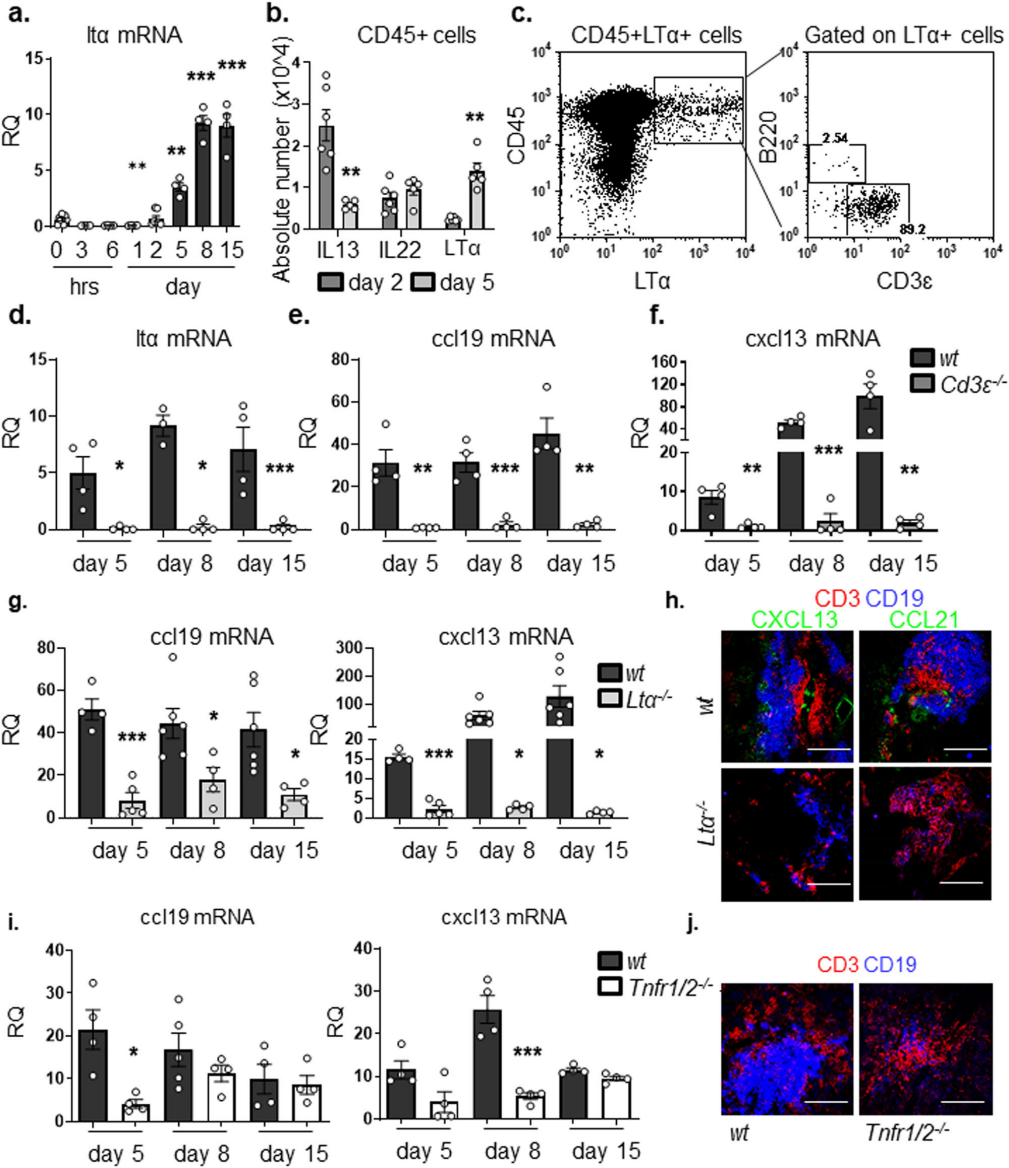
LTα3 is responsible for chemokine production within newly formed TLSs. **a** qPCR analysis of ltα mRNA transcripts from *wt* (black bars) salivary glands at day 0, 3 h, 6 h, day 1, day 2, day 5, day 8 and day 15 p.c. Gene expression was normalized to housekeeping gene β-actin and expressed as RQ values relative to day 0 mRNA transcripts results. **b** CD45+ cells expressing IL13, IL22 or LTα3 at day 2 p.c. (grey bars) and day 5 p.c. (white bars) in wt mice. **c** Enumeration of LTα + lymphocytes within the CD45+ cells in *wt* salivary glands at day 5 p.c. by flow cytometry. Representative dot plot of LTα expression in CD45+ cells and its frequency within CD3ε+ and B220+ cells. **d**–**f** Graphs showing qPCR analysis of ltα (**d**), ccl19 (**e**) and cxcl13 (**f**) mRNA transcripts from *wt* (black bars) and *Cd3ε*^*−/−*^ (dark grey bars) salivary glands at day 5, 8 and day 15 p.c. **g** Quantification of ccl19 and cxcl13 mRNA transcripts from *wt* (black bars) and *Ltα*^*−/−*^ (light grey bars) salivary glands at day 5, 8 and 15 p.c. In all cases, gene expression was normalized to housekeeping gene β-actin and expressed as RQ values relative to day 0 mRNA transcripts results. **h** Immunofluorescence staining in salivary glands from *wt* and *Ltα*^*−/−*^ mice for lymphoid aggregates with CD3ε (red), CD19 (blue) and CXCL13 or CCL21 (green) at day 15 p.c. Scale bar 100 µm. **i** qPCR analysis of ccl19 and cxcl13 mRNA transcripts from *wt* (black bars) and *Tnfr1/2*^*−/−*^ (white bars) salivary glands at day, 5, 8 and 15 p.c. Chemokine mRNA transcript results were normalized to β-actin. **j** Immunofluorescence staining for lymphoid aggregates within infected salivary glands from *wt* and *Tnfr1/2*^*−/−*^ mice with CD3ε (red) and CD19 (blue) at day 8 p.c. Scale bar 100 µm. Data are mean ± s.e.m from two independent experiments with three to six mice analyzed per group. *p < 0.05; **p < 0.01; ***p < 0.001, one-way ANOVA with Dunnett’s multiple comparison test (**a**) and unpaired *t* test (**b**, **d**, **e**, **f**, **g**, **i**).

We and others have previously demonstrated^8,10–12^ that LT family members regulate sustained chemokine production in assembling TLS. However, the signals responsible for LT production in TLS are not fully understood. Having induced TLS formation in wild-type (*wt)* murine salivary glands following intra-ductal delivery of replication-deficient adenovirus, we observed an increase in the gene expression of LTα and LTβ starting from day 5 p.c. onwards (Fig. 1a and Supplementary Fig. 1). Accordingly, enrichment for LTα3 producing CD45+ cells was observed at day 5 post TLS induction in comparison to IL13 which has been previously shown to play a role in TLS initiation (Fig. 1b and Supplementary Fig. 1b).

Within the CD45+ cells, T cells were found to be the predominant source of LTα3 in *wt* murine salivary glands (Fig. 1c and Supplementary Fig. 1). The specificity of our finding on LTα3 was confirmed by double surface and intracellular staining for lymphotoxin that confirmed the intracellular detection of LTα3 and not the surface expression of this protein, classically associate with membrane bound LTα1β2 in this early phase in T cells (Supplementary Fig. 1c). In order to confirm the role of CD3+ cells in early phases of TLS assembly we performed quantification of mRNA transcripts of infected salivary glands isolated from T-cell deficient mice (*Cd3ε*). We demonstrated a significant defect in LTα and LTβ transcripts, together with a significant decrease in the chemokines CCL19 and CXCL13, respectively required for migration and assembly of T and B lymphocytes into TLS (Fig. 1d–f and Supplementary Fig. 1).

We then aimed to evaluate the specific role of LTα3 in TLS assembly. We therefore induced TLS formation in *Ltα*^−/−^ and observed that TLS forming in *Ltα*^−/−^ exhibited a more severe phenotype than those forming in *Ltβr*^−/−^^8^, with a significant early defect in CCL19, CCL21 and CXCL13 expression (^8^ and Fig. 1g, h). In order to confirm that this defect was intrinsic to the stromal cells we isolated immunofibroblasts from *wt, Ltα*^*−/−*^ *and Ltβr*^*−/−*^ salivary glands 5 days post cannulation and we confirmed that the immunofibroblasts from *Ltα*^*−/−*^mice display a more profound defect in terms of chemokine production as compared to the *Ltβr*^*−/−*^ and the *wt* control (Supplementary Fig. 1).

Since LTα3 is known to signal through the TNF receptors (TNFR1/2) to induce chemokine expression, we postulated that salivary gland cannulation in the *p55/75*^*−/−*^ (defective for Tnfr1/2) mice would mirror the phenotype observed in *Ltα*^*−/−*^ and *Cd3ε*^*−/−*^ mice. First, we confirmed that immunofibroblasts express TNFR*α* and that this expression is upregulated post infections (Supplementary Fig. 1). We further confirmed that cannulated *p55/75*^*−/−*^ mice present an early defect both in CCL19 and CXCL13 transcript levels (Fig. 1i). Accordingly, immunofluorescence staining in the salivary glands of *p55/75*^*−/−*^ mice, revealed the presence of smaller lymphocytic aggregates with reduced T and B cell clusters (Fig. 1j). Interestingly, chemokine expression was not abrogated in the latest phases of TLS assembly, suggesting a compensatory mechanism in these knockouts (Fig. 1i).

### ICOS+ T cells are required for LTα3 expression in newly formed TLS

It has previously been shown that the co-stimulatory molecule ICOS along with LT signaling is required in a sequential manner for a GCs response in SLOs, and that ICOS is a prerequisite for LT expression on lymphocytes^53^. Interestingly, in our system, engagement of this pathway proceeds TLS and GC formation.

**Fig. 2:**
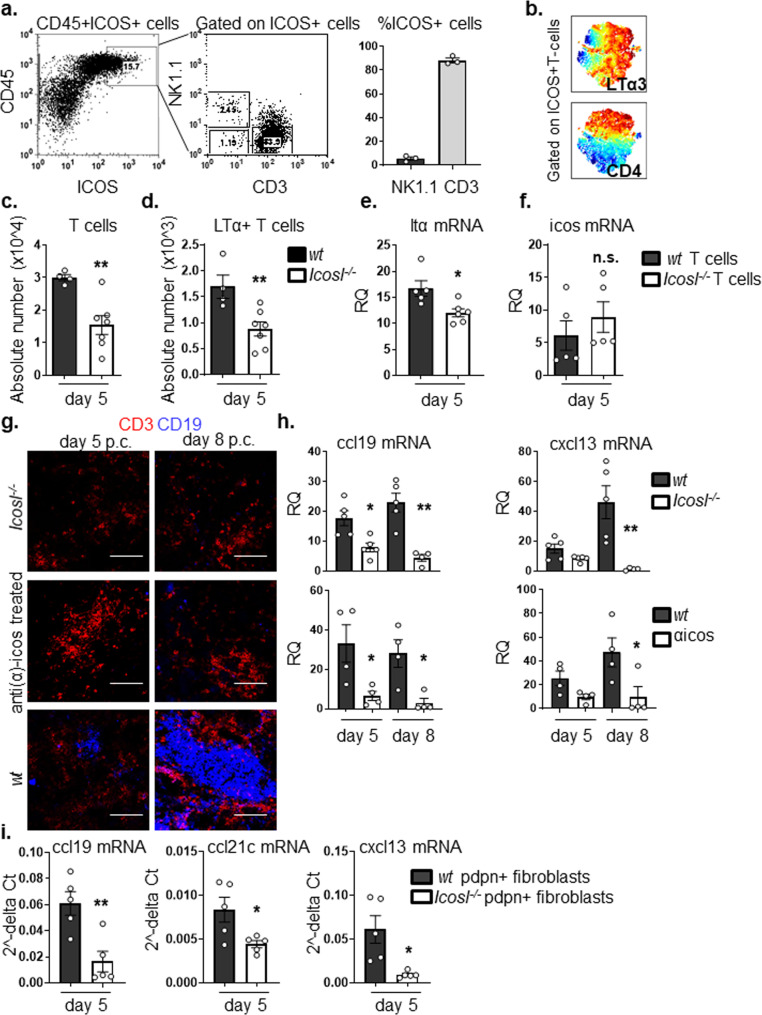
ICOS+ T cells regulate Ltα expression within newly formed TLSs. **a** Representative dot plots showing flow cytometry staining for ICOS expression in *wt* salivary glands at day 5 p.c. by CD45+ cells. Graph showing percentage of ICOS+ T lymphocytes and NK cells infiltrating salivary glands at day 5 p.c. **b** viSNE plots of ICOS+ T lymphocytes from infected salivary gland from *wt* mice at day 5 p.c., analyzed by multicolor flow cytometry. Colours indicate cell expression level of labelled markers (LTα3 and CD4). **c**, **d** Flow cytometry analysis of absolute numbers of T cells and LTα-producing T cells in *wt* (black bars) and *Icosl*^*−/−*^ (white bars) mice at day 5 p.c. **e**, **f** qPCR analysis of ltα and icos mRNA transcripts in FACS sorted CD3+ cells from *wt* (black bars) and *Icosl*^*−/−*^ (white bars) mice at day 5 p.c. mRNA transcripts were normalized to housekeeping gene β-actin and presented as RQ values calculated with calibrator day 0 CD45+ cells. **g** *wt* mice were left untreated or treated with anti-ICOS blocking antibody from day 0 post salivary gland cannulation and sacrificed at days 5 and 8 p.c. Cannulated *Icosl*^*−/−*^ were also sacrificed and analyzed at days 5 and 8 p.c. Representative microphotographs of salivary glands examined by immunofluorescence for CD3ε (red) and CD19 (blue) showing lymphocytic infiltration. Scale bar 100 µm. **h** qPCR analysis of mRNA obtained from salivary glands at day 5 and 8 p.c. of lymphoid chemokine ccl19 and cxcl13 in anti-ICOS treated *wt* mice or *Icosl*^*−/−*^ mice (both white bars) in comparison to *wt* mice (black bars). mRNA transcripts were normalized to β-actin mRNA and results are presented as relative quantitation (RQ). **i** Transcript levels for ccl19, ccl21c and cxcl13 in FACS sorted CD45-EpCAM-CD31-pdpn+ immunofibroblasts from *wt* (black bars) and *Icosl*^*−/−*^ (white bars) mice at day 5 p.c. Transcripts levels were normalised to β-actin. Data are mean ± s.e.m from two independent experiments with three to six mice analyzed per group. *p < 0.05; **p < 0.01; ***p < 0.001; n.s. non-significant, unpaired *t* test.

Incoming immune cells in the *wt* salivary glands already at day 5 post-viral delivery exhibited an activated phenotype and were characterized by expression of the co-stimulatory molecule ICOS. In addition to a small population of NK cell, the vast majority of ICOS+ cells were T cells (Fig. 2a and Supplementary Fig. 2, isotype control). ICOS is known to regulate T cell function, including cytokine production in different settings^54^. Indeed, the majority of ICOS+ T cells within inflamed salivary glands showed high expression for LTα3 (Fig. 2b).

In order to investigate whether the ICOS-ICOSL mediated co-stimulatory pathway was responsible for the production of LTα3 in TLS, we infected *Icosl*^*−/−*^ salivary glands and analyzed them at day 5 p.c. The salivary glands from the *Icosl*^*−/−*^ mice contained significantly lower T cell numbers (Fig. 2c). T cells in these mice showed both a numeric (Fig. 2d) and qualitative defect (Fig. 2e) in LTα production as compared to *wt* controls. However, ICOS expression on T cells was not impaired (Fig. 2f), and the defect in cytokine production observed was restricted to LTα, with the *Icosl*^*−/−*^ T cells displaying normal transcript levels for both LTβ and TNFα and a normal number of pdpn+ immunofibroblasts (Supplementary Fig. 2). Importantly, the number of LTα1β2 positive cells in the *Icosl*^*−/−*^ mouse was not significantly reduced (Supplementary Fig. 2), confirming our hypothesis that expression of LTα3 and not LTα1β2 is more critically affected by interference with this pathway.

Having identified a role for ICOS-ICOSL interaction in the upregulation of LTα, we predicted that interruption of ICOS signaling would also lead to a clear defect in TLS assembly. Indeed, we observed fewer T cells and minimal B cell influx, when TLS formation was induced in the salivary glands of either *Icosl*^*−/−*^ mice or *wt* animals treated with anti-ICOS blocking antibody (Fig. 2g). Similar to *Ltα*^*−/−*^ mice, the transcript levels of CCL19 were also significantly reduced when compared to controls both at day 5 and day 8 post TLS induction. On the contrary, CXCL13 appeared more significantly affected in the later phases of TLS development, suggesting differential regulation of these two chemokines in vivo by LTα (Fig. 2h). Interestingly, FACS sorted immunofibroblasts from *Icosl*^*−/−*^ mice showed significantly lower CCL19, CCL21c and CXCL13 expression when compared to their *wt* counterparts (Fig. 2i), despite the lack of difference in immunofibroblast cell numbers (Supplementary Fig. 2). These data suggest that interfering with the ICOSL-ICOS pathways results in a functional, rather than numerical, impairment of the immunofibroblast component.

### Immunofibroblasts represent an important source of ICOSL during TLS assembly

**Fig. 3:**
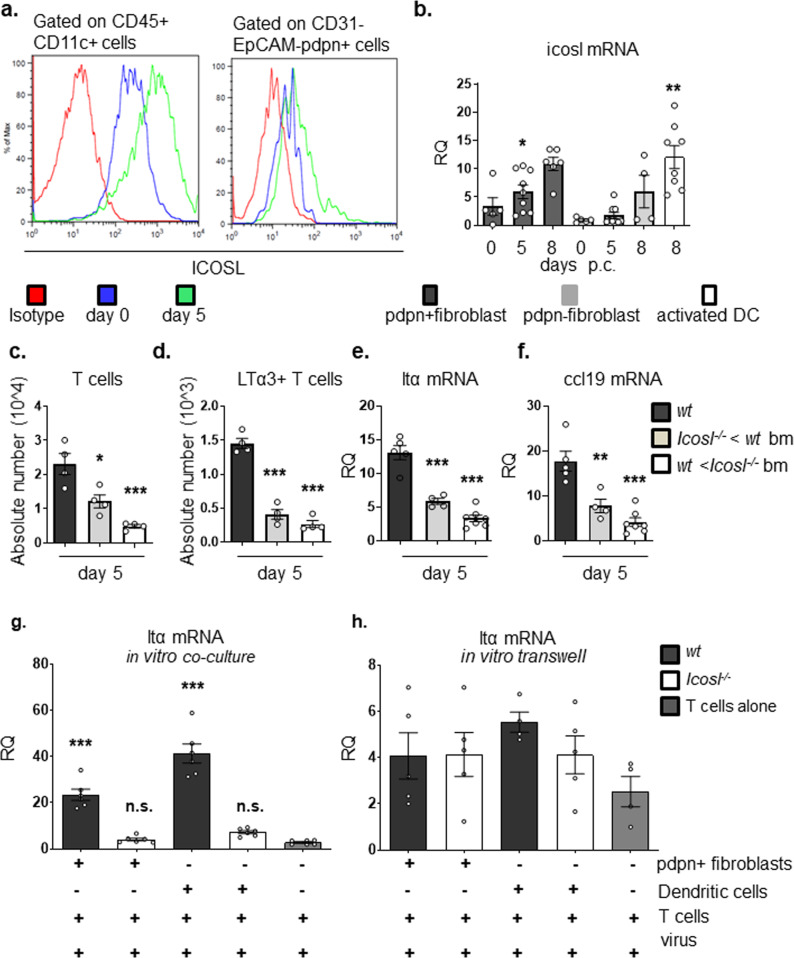
Immunofibroblast ICOSL contributes to TLS establishment. **a** Histogram showing flow cytometry staining for ICOSL expression on dendritic cells (CD45+ CD11c+ cells) and immunofibroblasts (CD45-EpCAM-CD31-pdpn+) in *wt* mice at day 0 (blue line) and day 5 p.c. (green line), and isotype control (red line). **b** *icosl* mRNA expression in FACS sorted CD45-EpCAM-CD31-pdpn+ immunofibroblasts (black bars), CD45-EpCAM-CD31-pdpn− fibroblasts (grey bars) at day 0, 5 and 8 p.c. and sorted activated dendritic cells (DCs) cells at day 8 p.c. (white bar). mRNA transcripts were assessed by qPCR and normalized to housekeeping gene β-actin. RQ values were calculated with calibrator day 0 pdpn- fibroblasts. **c**, **d** Absolute numbers of T cells (**c**) and LTα-producing T cells (**d**) in *wt* (black bars), *Icosl*^*−/−*^ mice reconstituted with *wt* bone marrow (grey bars) and *wt* mice reconstituted with *Icosl*^*−/−*^ bone-marrow (bm) (white bars) at day 5 p.c were determined by flow cytometry. **e**, **f** Quantitative real time PCR analysis of mRNA transcripts of *ltα* (**e**) and *ccl19* (**f**) in *wt* (black bars), *Icosl*^*−/−*^ mice reconstituted with *wt* bone-marrow (grey bars) and *wt* mice reconstituted with *Icosl*^*−/−*^ bone marrow (white bars). mRNA transcripts were normalized to housekeeping gene β-actin. **g**, **h** FACS sorted *wt* T cells were either co-cultured directly (**g**) or in trans-wells (**h**) with either *wt* or *Icosl*^*−/−*^ CD45-EpCAM-CD31-pdpn+ fibroblasts and dendritic cells (DCs) isolated at day 0 p.c. additionally stimulated with adenovirus. Graphs qPCR analysis of ltα mRNA transcripts normalized to the housekeeping gene β-actin are presented as relative quantitation (RQ) values calibrated to T cells cultured alone and the asterisks show significance as compared to T cells cultured alone. Data are mean ± s.e.m from two independent experiments with three to six mice analyzed per group. *p < 0.05; **p < 0.01; ***p < 0.001; n.s. non-significant, one-way ANOVA with Dunnett’s multiple comparison test (**b**, **g**, **h**) and one-way ANOVA with Tukey’s multiple comparison test (**c**–**f**).

In *wt* mice, we confirmed upregulation of ICOSL on classical antigen presenting cells, dendritic cells (Fig. 3a). Surprisingly, a significant enrichment of ICOSL transcript was also observed in the non-hematopoietic compartment and, in particular, on the same activated immunofibroblasts that were responsible for chemokine production (Fig. 3a, b and Supplementary Fig. 3 comparing with other cell populations). In order to investigate the relevance of this observation in vivo, we generated bone marrow chimeras of *Icosl*^*−/−*^ mice reconstituted with *wt* bone marrow (BM) and *wt* mice reconstituted with *Icosl*^*−/−*^ BM and studied TLS development as well as the regulation of LTα expression on T cells isolated from these mice. Flow cytometry analysis at day 5 post TLS induction demonstrated a significant reduction in the numbers of infiltrating T cells in both groups of chimeric mice when compared to *wt* controls (Fig. 3c). Additionally, the number of LTα-producing T cells and the expression of lymphoid chemokines associated with organization of the TLS were compromised when mice lacked ICOSL in either the hematopoietic or non-hematopoietic compartments (Fig. 3d–f and Supplementary Fig. 3). These findings suggest that both immune cells and immunofibroblasts provide ICOSL to incoming ICOS+ T cells (Fig. 3a, b).

To better dissect this co-stimulatory function of immunofibroblasts in vitro, we co-cultured TLS pdpn+CD31-EpCAM− immunofibroblasts or dendritic cells (DCs) isolated from either *wt* or *Icosl*^*−/−*^ mouse salivary glands with activated T cells isolated from *wt* spleen. Transcript analysis of T cells unveiled inability of T cells to upregulate LTα when cultured in direct contact with either immunofibroblasts or DCs isolated from *Icosl*^*−/−*^ mice compared to wt mice (Fig. 3g), recapitulating the effect observed in vivo in *Icosl*^*−/−*^ mice. Interestingly, this phenomenon was dependent on direct cell–cell contact, as the use of trans-well filters to separate T cells and either immunofibroblasts or DCs abolished this effect, with a 15-fold difference between LTα expressions in the trans-well filter model as compared to the direct co-culture setting for T cell cultured with ICOSL sufficient cells (Fig. 3h).

### ICOS-ICOSL interaction blockade in human minor and major salivary glands results in decreased LTα3 production by CD4 T cells

**Fig. 4:**
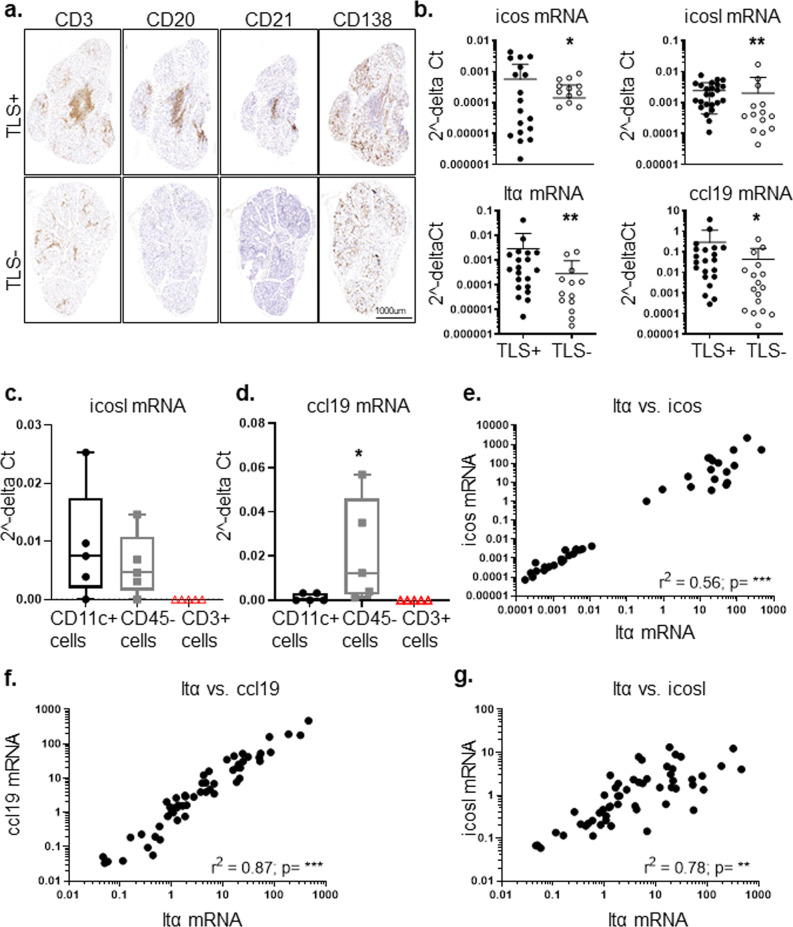
Expression of ICOS and ICOSL in the salivary glands of patients with Sjögren’s syndrome. **a** Representative minor salivary glands (mSGs) sequential sections were stained for CD3+ for T cells, CD20+ for B cells and CD138+ for plasma cells for histological identification of tertiary lymphoid structures (TLS) in Sjögren’s syndrome salivary glands. **b** qPCR analysis for mRNA transcripts of *icos, icosl, ltα* and *ccl19* in TLS+ (black dots, *n* = 27) and TLS− (open dots, *n* = 29) mSGs biopsies from Sjögren’s patients. mRNA transcripts were normalized to the housekeeping gene 18 S RNA transcripts. *p < 0.05; **p < 0.01, Mann–Whitney test. Data are representative as mean ± s.d. **c**, **d** Whisker plots showing icosl (**c**) and ccl19 (**d**) mRNA transcripts in dendritic cells (CD11c+), non-hematopoietic (CD45−) cells and T (CD3+) cells were sorted from primary Sjögren’s patients’ mSGs (*n* = 5). Data are presented as min to max, *p < 0.05, Mann–Whitney test. **e**–**g** Linear regression analysis confirms significant correlation of *ltα* mRNA with *icos* mRNA (**e**), *ccl19* mRNA (**f**) and *icosl* mRNA (**g**) in mSGs biopsies of Sjögren’s patients.

In order to validate the relevance of this pathway in human disease, we evaluated expression of ICOS and ICOSL in minor labial salivary gland biopsies from patients with Sjögren’s (SS) that display TLS (TLS + SS) in the salivary glands (Fig. 4a). Expression of ICOS, ICOSL, LTα and CCL19 transcripts were significantly increased in TLS + SS as compared to TLS − SS samples (Fig. 4b). ICOS expression was mainly detected on CD3+ cells; whereas ICOSL expression was detected within, but not limited to, the lymphocyte rich area in co-localization with CD20+ cells, CD11c+ cells and on podoplanin+ cells. RNAscope was used to confirm and quantify ICOSL distribution in the fibroblasts and DCs in the salivary glands (Supplementary Fig. 4). Expression of LTα was enriched in CD3+ cells (Supplementary Fig. 5) whereas ICOSL expression was detected in both sorted CD11c+ and CD45− cells (Fig. 4c) from salivary glands of SS patients. In contrast, CCL19 expression was confined to CD45− cells (Fig. 4d). Finally, LTα expression in human salivary glands showed a significant positive correlation with the expression of ICOS, CCL19 and ICOSL (Fig. 4e–g).

**Fig. 5:**
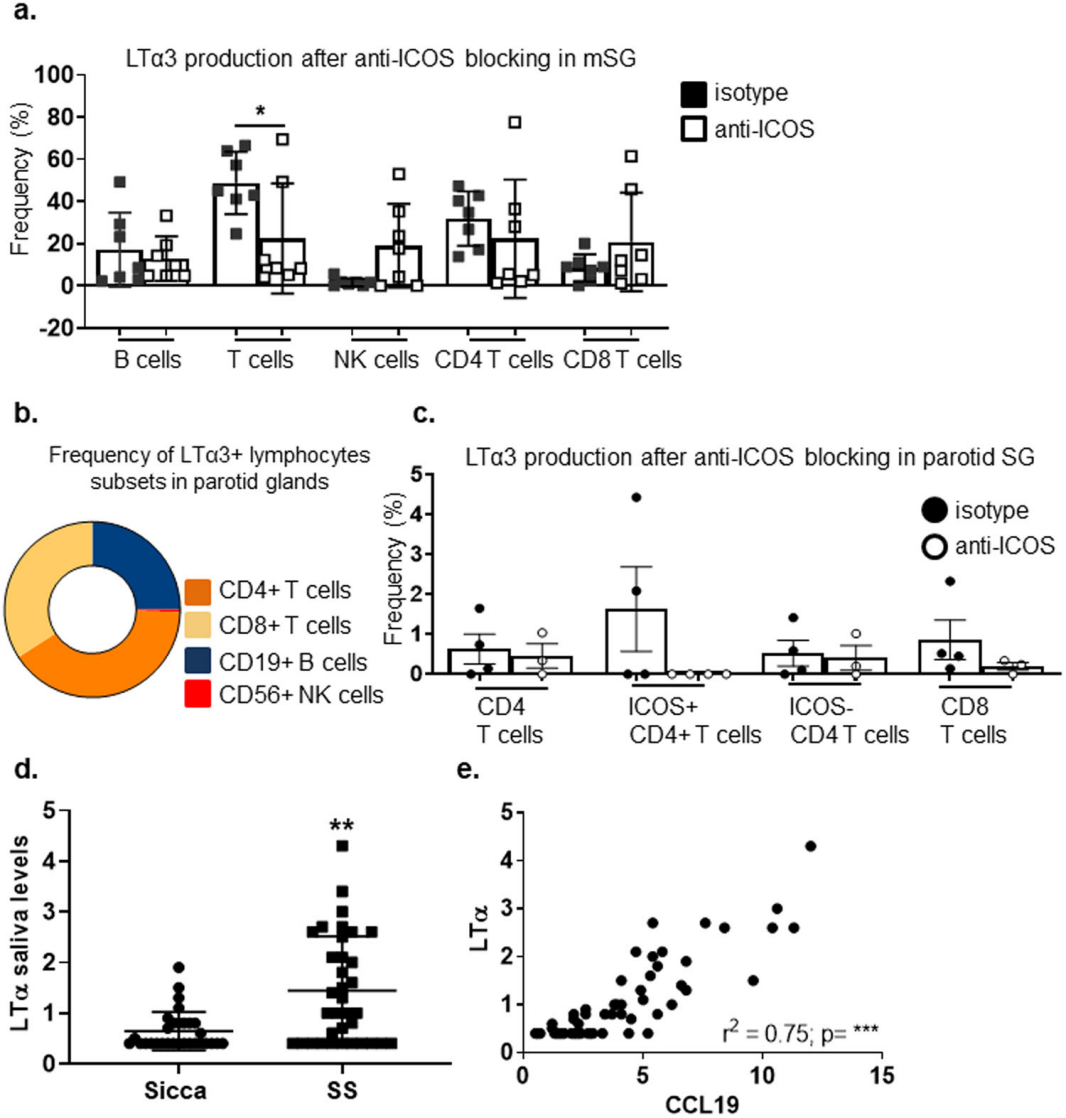
ICOS-ICOSL interaction influences LTα3 production in the salivary glands of patients with Sjogren’s syndrome and MALT lymphoma. **a** Graph showing frequency of LTα3 producing lymphocyte populations in mSGs biopsies (*n* = 7) treated with anti-ICOS (white squares) or as compared isotype antibody (black squares) treatment ex vivo. *p < 0.05, Mann–Whitney test. Data are representative as mean ± s.d. **b** Diagram showing LTα3 producing lymphocyte populations in MALT-parotid SG biopsies. **c** Graph showing percentage of LTα3 producing lymphocyte populations in MALT-parotid SG biopsy treated ex vivo with anti-ICOS (white circles) or as compared isotype antibody (black circles). Data are representative as mean ± s.d. **d** Graph showing saliva levels of LTα in sicca and Sjӧgren syndrome patients. **p < 0.01, Mann–Whitney test. Data are representative as mean ± s.d. **e** Graph showing linear regression analysis to confirm correlation between LTα and CCL19 detected in saliva of Sjӧgren syndrome.

In order to confirm the functional relevance of this pathway in humans, and evaluate the requirement for this pathway in established disease, we treated minor salivary gland explant tissue from patients with SS and a major salivary gland from a patient diagnosed with a low grade mucosal associated lymphoid tissue (MALT) lymphoma with an anti-human ICOS blocking antibody (or isotype control) in vitro and assessed LTα production in the lymphocyte subsets as described above for the murine cells. ICOS blockade, in vitro, was associated with a decrease LTα production in CD4+ T cells in both settings (Fig. 5a–c), suggesting the validity of our finding on the requirement of the ICOS /ICOSL pathway in the context TLS formation in humans.

Interestingly, analysis of stimulated saliva samples from SS and sicca patients for inflammation-related protein biomarkers using Proseek Multiplex INF^96*x*96^ revealed that sixteen proteins in saliva were significantly separated between SS and sicca patients, LTα levels exhibited an independent and significant association with SS in both univariate and multivariate analysis (Fig. 5d and Supplementary Tables 1, 2). Levels of saliva LTα were strongly correlated with saliva CCL19 (r = 0.7475; *p* < 0.0001) (Fig. 5e). Although, we observed higher levels of LTα in the serum of SS patients as compared to sicca patients, it could not classify SS versus sicca using univariate or multivariate logistic analysis. Furthermore, there was a lack of correlation observed between saliva and serum LTα protein levels suggesting that saliva LTα is a strong indicator of local salivary gland inflammation as opposed to serum LTα (Supplementary Fig. 6). Together, these data suggest that saliva but not serum LTα levels could be utilized to identify the local inflammatory process with potential diagnostic/prognostic value.

## Discussion

**Fig. 6:**
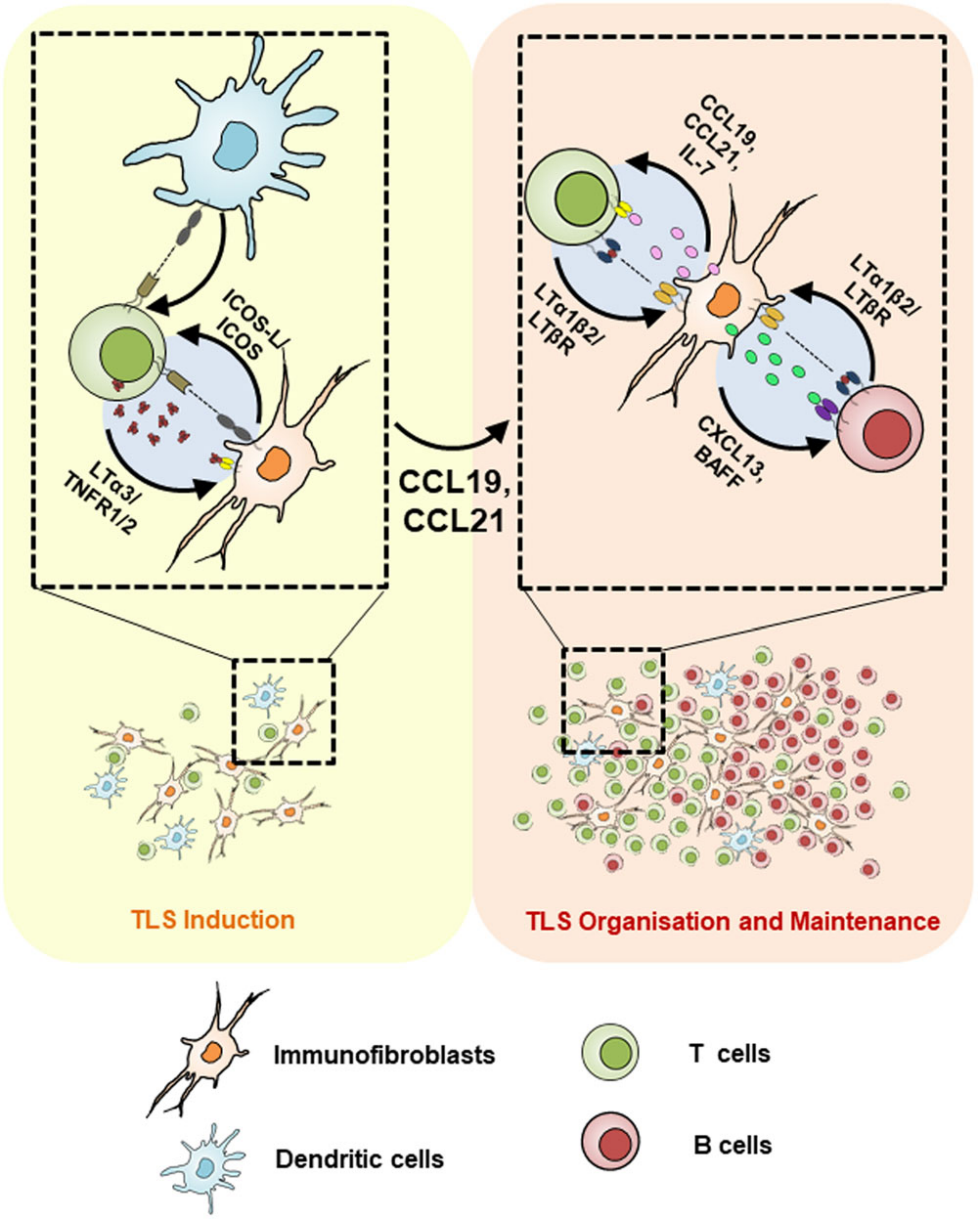
Model of immunofibroblast- T cell cross-talk via ICOSL/ICOS engagement in TLS formation. During inflammation in a non-lymphoid organ, activated resident immunofibroblasts and DCs upregulate ICOSL, which interacts with the locally recruited ICOS+ T cell contributing to their secretion of LTα. Engagement of LTα3 with TNFR1/2 on immunofibroblasts induces CCL19 and CCL21 chemokine production that is crucial for the positive feedback loop involved in TLS formation.

Co-stimulation is the process by which immune cells receive micro-environmental cues to shape an immune response. Here we demonstrate for the first time that, within TLS, immunofibroblasts, alongside classical antigen presenting cells, are able to provide key co-stimulatory signals to incoming lymphocytes, enabling the production of LTα3 which, in turn, stimulates the same immunofibroblasts to produce chemokines required for lymphocyte accumulation. This amplificatory loop is mediated by the co-stimulatory pair ICOS/ICOSL and is responsible for the switch from transient activation to the full establishment of the functional TLS immunofibroblast network mediated by the lymphotoxin family members (Fig. 6). Thus, we reveal, for the first time, a critical co-stimulatory role for immunofibroblasts in driving TLS formation.

The relative contribution of LTα1β2 (via LTβR engagement) compared to LTα3 (via TNFR1/2) in the development and adult maintenance of SLO is well established^13–17,21,24–28,54^. In contrast, very little is understood about the relative role played by these cytokines during TLS assembly.

We and others, in separate models of TLS, have demonstrated that LTβR signaling drives functional immunofibroblast maturation, which comprises stable chemokine and survival factor expression^5,8,10–12,52^. In our model of salivary gland inflammation, absence of *Ltβ* signal via *Ltβr* results in marked impairment in CXCL13 production and failure to recruit B cells^8^. Nonetheless, early production of CCL19 is maintained, suggesting that the expression of CXCL13 and CCL19 is differentially regulated in TLS. Here, we demonstrate that *Ltα*^*−/−*^ mice displayed a more severe impairment in both CXCL13 and CCL19 expression and a complete abolishment of the TLS establishment as compared to the phenotype observed in the *Ltβr*^*−/−*^ mice^8^. The absence of LTα severely impairs early T cell recruitment, suggesting that the LTα3-TNFR1/2 signaling axis regulates early CCL19 and CCL21 production in a non-redundant manner; this phenotype was confirmed in *p55/75*^*−/−*^ (defective in TNFR1/2) mice. Interestingly, the phenotype observed in the *p55/75*^*−/−*^ was not as severe as that observed in the *Ltα*^*−/−*^ mice, suggesting that LTβR signalling collaborates with TNFR1/2 for chemokine production in immunofibroblasts. Similarly, Vu et al. previously demonstrated that GC B cells from *Icos*^*−/−*^ mice express lower levels of LTα1β2 compared to wild-type GC B cells in vivo; interestingly, in that setting, the administration of agonistic anti-LTβ receptor antibody was unable to restore the GC response, also suggesting that additional input from another pathway or engagement of a different receptor is required to accomplish GC generation^53^. LTα over-expression, under the control of rat insulin promoter (RIP), is known to induce TLS formation in a process that is dependent on TNFR1, but not LTβR, confirming that signals through TNFR are necessary and sufficient for the initiation of TLS development^48–50^. Moreover, engineered LTα expression in tumour cells leads to the formation of intra-tumoral lymphoid tissue, able to sustain an efficient immune response, suggesting that signalling through LTα and its receptor plays a role in the induction of functionally competent TLS^55^. However, mice overexpressing both LTα and LTβ display much larger infiltrates with better segregated T and B cell areas and higher expression of lymphoid chemokines than those observed in the LTα single transgenic mice^51^. All together, these findings suggest that, while LTα3 plays a non-redundant role in TLS initiation, the synergistic activation of TNFR1, TNFR2 and LTβR is required for the full development of TLS.

It has been previously demonstrated that, during TLS ontogeny, T cells can recapitulate the role played by Lymphoid Tissue inducer cells that express LT during SLO organogenesis^8,11,12,52,56,57^. In this study, we demonstrated that, in absence of adequate levels of T cell-secreted LTα, immunofibroblasts are not able to produce the lymphoid chemokines required for the early stage of TLS assembly, providing a mechanistic link between T cells, lymphotoxin expression and early chemokine production within TLS. In our model, CCL19 is more susceptible to the decrease in LTα, as compared to CXCL13. Indeed, while CXCL13 expression can be observed in several conditions, not necessarily characterised by lymphoneogenesis such as mycobacteria granuloma^58^, the expression of both CXCL13 and CCL19 in a regulated and organised manner is critically associated with TLS development^59^. In this context, the earlier expression of LTα regulates CCL19 production by local immunofibroblasts might help defining the anatomical site for TLS assembly.

Lymphotoxin production by activated T cells has been previously reported, in association with engagement of co-stimulatory molecules alongside TCR signaling^60,61^. Here we show that immunofibroblasts act in concert with classical antigen presenting cells, providing ICOSL to incoming lymphocytes. In absence of this signal, T cells fail to produce enough LTα3 to support CCL19 production.

The relative contribution of different co-stimulatory pathways within TLS have not been widely considered. However, a significant role for ICOS could be predicted based on its role in the activation, maintenance, and function of activated/effector T cell populations and in the formation of fully functional germinal centers^54^. Importantly ICOS signaling induces and maintains T follicular helper cells (Tfh), drives B cell affinity maturation and plasma cell differentiation within GCs^62–66^. Accordingly, pharmacological or genetic interference with the ICOS pathway prevents the development of spontaneous disease in pre-diabetic *NOD* mice^67^ and ameliorates both collagen-induced arthritis and systemic lupus erythematous^68,69^. Here we demonstrate that the role of this signaling pathway exceeds these previously described functions, positioning the ICOS/ICOSL interaction at the initiation phases of TLS assembly and not only in the latest phases of the GC response. Our work highlights a key role for this pathway, and an unexpected source for ICOSL in activated immunofibroblasts, alongside classical antigen presenting cells such as dendritic cells, revealing a critical link between ICOS and LTα in TLS mediated pathology.

Importantly, we provide evidence that this pathway might be active in human TLS, both in inflammatory conditions and in the earliest phases of malignant transformation (in the MALT lymphoma parotid treated in vitro with ICOS blocking antibody), where ICOS/ICOSL activation is continuously required for LT production and the aberrant lymphoid chemokine/cytokine expression. Interestingly, in addition to its pathogenic role, this pathway can be exploited as a biomarker of disease as we showed that LTα expression correlates with CCL19 levels and distinguishes patients with autoimmunity and dryness (Sjögren’s syndrome) from patients with dryness only (sicca patients). Whilst we cannot exclude that this pathway might be relevant in other models of inflammation, in our model it appears clear that ICOS-LTα-CCL19 axis represents a defining molecular signature of TLS that can be utilized as a diagnostic indicator for patient stratification and precision medicine.

Recent publications highlighted the important role of TLS in the context of cancer development and response to immunotherapy treatment^70–72^. The implementation of serum biomarkers such as LTα might represent an important tool in this context to stratify patients and further research on the prognostic value of these indicators of TLS formation in cancer patients should be fostered.

In summary, our data establish a novel functional connection between co-stimulatory molecules present on immunofibroblasts and other antigen presenting cells, and their cognate receptors on immune cells, unveiling a novel role for a classical co-stimulatory pathway in TLS associated disease and an unexpected immuno-regulatory role for immunofibroblasts in the context of chronic inflammation. These data, while supporting the development of therapeutic applications aimed at blocking co-stimulatory molecules in general and the ICOSL/ICOS pathway add to the growing understanding of the role of non-immune cells in the context of tissue pathology in chronic inflammation and cancer.

## Methods

### Mice and salivary gland cannulation

C57BL/6 wild-type *(wt)* mice were purchased from Charles River. *Ltα*^*−/−*^ and *Tnfr1/2*^*−/−*^ (p55/75) mice were provided by Dr J. Caamaño; *Cd3ε*^*−/−*^ (Tg(CD3ε)26Cpt) and *Icosl*^*−/−*^ mice were provided by Dr D. Withers (University of Birmingham, UK). To generate bone marrow chimeras, C57BL/6 and *Icosl*^*−/−*^ mice were sub-lethally-irradiated (2 × 450 Rad), given bone-marrow cells intravenously from *Icosl*^*−/−*^ and C57BL/6 mice respectively and used 4–5 wks after reconstitution. All mice were maintained under specific pathogen-free conditions in the Biomedical Service Unit at the University of Birmingham according to Home Office and local ethics committee regulations. Under ketamine/domitor anaesthesia, the submandibular glands of female C57BL/6 and knock-out mice (8–12 weeks old) were intra-ductally cannulated with 10^8^–10^9^ p.f.u. of luciferase-encoding replication-defective adenovirus (Adv5) as previously described^8,46,52^.

### ICOS antibody injection

Mice were injected i.p. at 10 mg/kg with anti-ICOS blocking antibody^73^ every 4 days starting from day 0 p.c.

### Human salivary gland biopsies, saliva and serum from Sjögren’s Syndrome (SS) patients

Minor salivary gland (mSGs) samples, saliva and serum were obtained from patients recruited in the OASIS cohort (Optimising assessments in Sjögren’s syndrome) at University Hospitals Birmingham NHS Trust. Saliva was obtained by the patient rolling a sterilized marble around their mouth over a 5-minute period. All subjects provided written informed consent and the study was approved by the Wales Research Ethics Committee 7 (WREC 7) formerly Dyfed Powys REC; 13/WA/0392.

Specimens were identified among samples obtained from by patients diagnosed with SS according to the 2016 ACR/EULAR classification and fulfilling the histological criteria for the diagnosis of SS (presence of focus score > 1). All patients included were untreated with immunosuppressive drugs including steroids. Minor and major SG from SS patients recruited at Barts Health NHS Trust in London were also used in the study. All subjects provided written informed consent and SGs were collected under ethics number: REC 05/Q0702/1 for Rheumatology/Oral medicine clinic-Barts Health NHS Trust.

Sicca samples were selected from patients having undergone investigation for SS, because of clinical symptoms of dryness (eyes and/or mouth), but who did not fulfil the classification criteria for SS, were not clinically diagnosed as primary or secondary SS by the leading physician and were anti-Ro negative.

### Histological identification of TLS in the salivary glands of Sjogren’s syndrome patients

Histological characterization of TLS+ and TLS− salivary glands was performed as previously described in^74^. Briefly, salivary gland sequential sections were stained for CD3 for T cells, CD20 for B cells, and CD138 for plasma cells. The staining amount of each antigen was scored with a semi-quantitative method. The scores ranged from 0 to 3, according to the inflammation degree, where a score of 0 corresponded to no cell infiltration, scattered inflammatory cells to 1, clusters of lymphocytes organized around the excretory ducts to 2 with one aggregate and 3 with more than one aggregate. Salivary glands infiltrates with T cells and B cells infiltration score ≥ 2, as well as showing B/T cell segregation and surrounded by plasma cells were classified as ectopic lymphoid structures (TLS).

### Histology and immunofluorescence of murine salivary glands

This was performed as previously described in^8,52^. Briefly, salivary glands from mice were harvested, snap frozen in OCT. Frozen sections of 6 µm in thickness were cut, left to dry overnight at room temperature and stored in −80 °C until use. For immunofluorescence analysis, slides were allowed to reach room temperature and then fixed for 20 min in ice-cold acetone, left to dry and then were hydrated in PBS. For immunofluorescence (IF) staining, all dilutions of reagents and antibodies were made in PBS with 1% BSA (Sigma). Firstly, the sections were treated with sequential avidin-biotin treatment to block endogenous biotin, sections for 15 min each and washed for 5 min with PBS in between the two incubations, followed by blocking with 10% horse serum for 10 min. Slides were then incubated for 60 min with primary antibodies followed by incubation with secondary antibodies for 30 min. Hoechst (Molecular Probes) was used for nuclear stain. Slides were mounted with Prolong Gold Antifade reagent (Invitrogen Life Technologies).

Images were acquired on a Zeiss LSM 510 laser scanning confocal head with a Zeiss Axio Imager Z1 microscope. Digital images were recorded in four separately scanned channels with no overlap in detection of emissions from the respective fluorochromes. Confocal micrographs were stored as digital arrays of 2048 × 2048 pixels with 8-bit sensitivity; detectors were routinely set so that intensities in each channel spanned the 0–255 scale optimally. The LSM510 Image Examiner Software was used to process these images.

### Immunohistochemistry

Formalin-fixed, paraffin-embedded (FFPE) labial salivary gland biopsies from Sjögren’s syndrome patients were processed for staining as previously described in^39^. For incubation of primary antibodies, amplification methods and chromogens, the EnVision^TM^ G | 2 Doublestain System, Rabbit/Mouse (DAB+/Permanent Red) (Dako) was used according to manufacturer’s instructions. This system allows the simultaneous detection of two different antigens in one section; the first antigen is identified with peroxidase (HRP) and DAB as the chromogen, and the second with alkaline phosphatase (AP) and Permanent Red. In brief, sections were blocked with Dual Endogenous Enzyme Block for 5 min, washed in TBS and the first primary antibody (ICOS clone SP98 Novus Biologicals, podoplanin clone D2-40 AbD Serotec or ICOSL LSBio) diluted 1:10 in TBS (+1% BSA) was incubated for 1 h at room temperature. The secondary antibody with Polymer/HRP was incubated for 10 min and slides washed twice with TBS. Incubation with DAB took place under the light microscope and the reaction was stopped with distilled water when optimal brown staining was achieved. Followed by a blocking step with Doublestain Block reagent (3 min), the sections were incubated with the second diluted primary antibody (CD3 polyclonal rabbit (1:80) Dako, CD20 clone L26 (1:20) Dako, CD11c clone 5D11 (1:10) Leica or ICOSL (1:10) LSBio) for 1 h. The following step was the Rabbit/Mouse (LINK) for 10 min and then another 10 min with Polymer/AP. The second reaction was then developed with Permanent Red and once again it took place under the light microscope until optimal red staining was achieved. Sections were dehydrated in increasing concentrations of ethanol (80%, 95 and 100%) and placed in xylene for five min, twice. Finally, slides were mounted with Mounting Medium DPX (Sigma–Aldrich) and scanned using a Zeiss AxioScan.Z1 slide scanner.

### RNAscope

Indirect detection by fluorescence was based on the Opal^TM^ Multiplex IHC method (Akoya), and performed on the Autostainer Leica Bond RX. Samples were probed for RNAscope^®^ LS 2.5 Probe - Hs-ICOSLG - Homo sapiens inducible T-cell co-stimulator ligand (ICOSLG) transcript variant 1 mRNA or Negative control probe DapB (of Bacillus subtilis strain) following manufacturer’s instructions (ACDBio). Subsequent staining was performed with antihuman CD11c (Abcam) and PDPN (Abd Serotech) antibodies. Staining was performed using the Opal^TM^ 7-Color Automatic IHC Kit (Akoya) according to the manufacturer’s recommendations. Signal amplification and covalent binding of fluorophore was achieved by using a tyramide signaling amplification reagent (included in the Opal kit) that is conjugated with a different fluorophore for each cycle. Multispectral images were acquired at ×20 magnification using the Vectra Polaris Automated Quantitative Pathology Imaging System (Akoya). MoTIF settings were used for multispectral image acquisition. Multispectral image processing of multiplex IHC stains was performed using Phenochart (version 1.0.11/Akoya) and inForm Image Analysis Software (version 2.3, Akoya).

### Isolation of murine stromal cells, leukocytes, in vitro stimulation for cytokine production and flow cytometry

These were performed as previously described in^8,52^. Concisely, for stromal cells salivary glands were cut into small pieces and tissue digested for 40 min at 37 °C with gentle stirring in 1.5 ml RPMI 1640 medium (Sigma) containing collagenase D (3.7 mg/ml; from Roche), DNase I (30 ug/ml; from Sigma) and 2% (vol/vol) fetal calf serum (FCS). Followed by further digestion for 20 min at 37 °C with medium containing collagenase dispase (3.7 mg/ml; from Roche) and DNase I (30 ug/ml). Leukocyte isolation was performed by salivary gland digestion for 20 min at 37 °C with gentle stirring in 2 ml RPMI 1640 medium containing collagenase dispase (250 ug/ml), DNase I (25 ug/ml) and 2% (vol/vol) FCS. Briefly, single-cell suspensions were incubated with the surface antibodies mix (Supplementary Table 3) for 30 min. Intracellular staining for cytokine production was performed (BD Cytofix/Cytoperm) according to the manufactures protocol and stained for 1 h with intracellular antibodies mix or isotype control (Supplementary Table 3). Stained cells were acquired using a Cyan-ADP (Dako) or LSRFortessa (BD). Data were analyzed with FlowJo software (Tree Star). For cell sorting, stained cells were sorted using MoFlo-XDP (Beckman Coulter Inc). The purity of sorted stromal populations routinely exceeded 96%.

#### In vitro LTα cytokine stimulation assay

Isolated stromal cells or dendritic cells were re-suspended at the same cell density in 500 µl of DMEM medium (Sigma) (with 10% FCS, 1%GPS, 1% NEAA, 1%HEPES and 50 µM β-mercaptoethanol) for in vitro LTα cytokine stimulation assay in 48 well plates. T cells isolated from spleen of wt mice were incubated with 50 ng/ml PMA, 750 ng/ml Ionomycin and 10 ul of 10^8^–10^9^ p.f.u. of adenovirus for 4 h at 37 °C. Stimulated cells T cells were then added at 1:4 ratios to 48 well plates with stromal cells and dendritic cells with or without virus. Cells were harvested after 24 h and taken for quantitative PCR analysis.

#### Ex-vivo assay and flow cytometry on human salivary glands

MALT- parotid gland tissue and minor salivary glands lobules were cultured in complete RPMI medium, for 2, 3 and 5 days. Cells egressed from the tissues were collected and stimulated with PMA (50 ng/mL), ionomycin (750 ng/mL) and BrefeldinA (10 μg/mL) for 4 h at 37 °C. MALT- parotid gland tissue and minor salivary glands lobules were cultured in complete RPMI medium (10% FBS, Penicillin/ streptomycin antiobiotics) for 2, 3 and 5 days or incubated with either anti-ICOS blocking antibody (clone JTA-009)^75,76^ or its isotype control (10 μg/mL), for 5 days. Cells egressed from the tissues and cells obtained from the digestion of the leftover of the tissue used for organ culture were collected and stimulated with Phorbol-12-Myristate-13-Acetate (final concentration 50 ng/mL), ionomycin (final concentration 750 ng/mL) and BrefeldinA (final concentration 10 μg/mL) for 4 h at 37 °C.

After Fc-block and viability dye staining (Biolegend), single-cell suspensions were incubated with the surface antibodies mix (Supplementary Table 4) for 20 min. For intracellular staining of Ltα, cells were permeabilized using the permabilisation kit (eBioscience) and stained for 1 h with intracellular antibodies mix or isotype control (Supplementary Table 4). Experiments were acquired within 24 h using a LSR-Fortessa (Becton Dickinson). For absolute count analysis, CountBright beads (LifeTechnologies) were added to the samples. Data were analysed with FlowJo vX software (Flowjo LLC).

### RNA isolation, reverse transcription and quantitative PCR for mouse salivary gland whole tissues and isolated cells

Quantitative real-time PCR was performed as previously described in^8,52^ using following primers Ccl19 (Mm00839967_g1), Cxcl13 (Mm00444533_m1), Lta (Mm00440229_g1), Ltb (Mm00434774_g1), Tnf (Mm00443258_m1), Tnfrsf1a alias Tnfra (Mm00441883_g1), Icos (Mm00497600_m1), Icosl (Mm00497237_m1) and Actb alias β-actin (Mm01205647_g1) was used as an endogenous control. Briefly, total RNA was isolated from salivary glands with an RNeasy mini kit (Qiagen) and the RNA was then reverse-transcribed using the high capacity reverse transcription cDNA synthesis kit (Applied Biosystems) according to the manufacturer’s specifications. Reverse transcription was carried out on Techne 312 Thermal Cycler PCR machine. Quantitative RT-PCR (Applied Biosystems) was performed on cDNA samples with the indicated primers and was run in duplicates on a 384-well PCR plate (Applied Biosystems) and detected using an ABI PRISM 7900HT instrument. Results were analyzed with the Applied Biosystems SDS software (SDS 2.3).

#### RNA isolation, reverse transcription and quantitative PCR for mSGs from human SS patients

Total RNA from minor salivary gland biopsies of Sjögren’s Syndrome patients (TLS+ and TLS− groups) and sicca syndrome was extracted using Trizol (Invitrogen) and the Direct-zol kit (Zymo research). Complementary-DNA was synthesised using the SuperScript IV Reverse Transcriptase kit (Thermofisher scientific) and diluted to 5 ng/μl using RNAse-free water. Quantitative real-time PCR was performed on a 7900HT Fast Real-Time PCR System (Applied biosystems) using TaqMan Gene Expression Master Mix (Applied biosystems) and the following primers: ICOS (Hs00359999_m1), ICOS-L (Hs00323621_m1), LTA (Hs04188773_g1) and CCL19 (Hs00171149_m1). All samples were run in duplicate and replicates with more than 2 cycles between them were discarded. Gene expression data was analysed using the 2^−delta Ct^ method using ribosomal 18 S RNA (Hs99999901_s1) as housekeeping gene.

#### Statistic and reproducibility

Statistics were performed with Prism software (GraphPad software Inc, San Diego, CA) using a Mann–Whitney test, student’s *t* test and one-way ANOVA as applicable. For correlation analysis, Spearman rank coefficient ‘r’ was calculated.

For data in Fig. 5d, e and Supplementary Fig. 6, descriptive summary statistics were generated to determine the baseline characteristics of the study population. After assessing the normal distribution of values (Shapiro–Wilk test), data were summarized as median and 25th–75th percentile. Salivary biomarkers concentration differences between groups were evaluated using the Wilcoxon rank-sum test or Chi-squared test, as appropriate. The Pearson’s test was used to explore the relationship between CCL19 and LTα saliva concentration. Univariate and multivariate logistic analysis was performed in order to reveal significant predictors of disease. Statistical analysis was performed using the R statistical software (version 3.5.0, The R Foundation for Statistical Computing, Vienna, Austria).

## Supplementary information

Supplementary Information

Description of Additional Supplementary Files

Supplementary Data 1

## Data Availability

The data supporting this study are available in the Article. Source data are available in Supplementary Data 1.
