# Supplementary material for: Immunofibroblasts regulate LTα3 expression in tertiary lymphoid structures in a pathway dependent on ICOS/ICOSL interaction
Source: Commun Biol. 2022 May 4;5:413. doi: 10.1038/s42003-022-03344-6 (PMC9068764; doi:10.1038/s42003-022-03344-6)
Supplement: Supplementary file 1 — Supplementary Information [file 42003_2022_3344_MOESM1_ESM.pdf]

Manuscript: Immunofibroblasts regulate lymphotoxin alpha 3 expression in tertiary lymphoid structures in a pathway dependent on ICOS/ICOS-L interaction.

SUPPLEMENTARY INFORMATION

Supplementary Figure Legends

Supplementary Fig. 1. LTβ expression within salivary glands during TLS formation. (a) qPCR analysis of ltβ mRNA transcripts from wt (black bars) salivary glands at day 0, 3hours, 6 hours, day 1, day 2, day 5, day 8 and day 15 p.c. Gene expression was normalized to housekeeping gene β-actin and expressed as RQ values relative to day 0 mRNA transcripts results. (b) Representative dot plot of flow cytometry isotype control staining for LTα expression by CD45+ cells in wt and Ltα-/- salivary glands at day 5 p.c. (c) Representative dot plot of flow cytometry staining for surface (S) and intracellular (IC) LTα expression in wt glands at day 5 p.c. (d) qPCR analysis of ltβ mRNA transcripts from wt (black bars) and Cd3ε -/- (dark grey bars) salivary glands at day 5, 8 and day 15 p.c. Gene expression was normalized to housekeeping gene β-actin and expressed as RQ values relative to day 0 mRNA transcripts results. (e) qPCR analysis of ccl19, ccl21 and cxcl13 mRNA transcripts in pdpn+ fibroblasts from wt (black bars), Ltβr-/- (blue bars) and Ltα -/- (grey bars) salivary glands at day 5 p.c. Gene expression was normalized to housekeeping gene β-actin. (f) tnfrα mRNA expression in FACS sorted CD45-EpCAM-CD31-pdpn+ immunofibroblasts at day 0, 5, 8 and 15 p.c. mRNA transcripts were assessed by quantitative real time PCR and normalized to housekeeping gene βactin. RQ values were calculated with calibrator day 0 CD45-EpCAM-CD31-pdpn+ fibroblasts. Data are mean ± s.e.m from two independent experiments with four to six mice analyzed per group. ** $p < 0.01$; *** $p < 0.001$, one-way ANOVA with Dunnett's multiple comparison test (a and f), one-way ANOVA with Tukey's multiple comparison test (e) and unpaired t test (d).

Supplementary Fig. 2. Other lymphoid cytokines and immunofibroblast induction are intact in Icosl-/- mice. (a) Representative dot plot of flow cytometry isotype control staining for ICOS expression by CD45+ cells in wt salivary glands at day 5 p.c. (b) qPCR analysis of mRNA transcripts ltβ and tnfα in CD3+ cells from wt (black bars) and Icosl-/- (white bars) mice at day 5 p.c. Transcripts were normalized to β-actin and expressed relative to day 0 CD45+ results. (c) Graph showing absolute number of podoplanin (pdpn)+ immunofibroblasts in day 5 p.c. salivary glands of wt (black bars) and Icosl-/- (white bars) mice. (d) representative flow cytometry plots showing LTβ staining within CD3+

of wt (black bars) and Icosl-/- (white bars). Data are mean ± s.e.m from two independent experiments with four to six mice analyzed per group. n.s. non-significant, unpaired t test.

Supplementary Fig. 3. Immunofibroblasts in TLS express ICOSL. (a) Graph showing absolute number and percentage of ICOSL+ cells in different cell populations (pdpn+ immunofibroblasts, pdpn- cells, CD11c+ dendritic cells, EpCAM+ epithelial cells and CD31+ endothelial cells) within a wt salivary gland at day 5 p.c. (b) Quantitative real time PCR analysis of cxcl13 mRNA in wt (black bars), Icosl-/- mice reconstituted with wt bone marrow (grey bars) and wt mice reconstituted with Icosl-/- bone marrow (white bars). mRNA transcripts were normalized to housekeeping gene β-actin. Data shown are mean ± s.e.m of two independent experiments with three to five mice analyzed per group. * $p < 0.05$; ** $p < 0.01$, one-way ANOVA with Tukey's multiple comparison test (b).

Supplementary Fig. 4. ICOS/ICOSL expression within salivary glands of Sjögren's patients. (a) Representative flow cytometry plots for ICOS expression in T lymphocytes and its distribution within CD4 and CD8 T cells within mSGs of Sjögren's patients. Isotype control staining is also shown. (b-d) Representative microphotographs of human salivary glands of patients diagnosed with Sjögren's syndrome double stained with anti ICOS (brown in b) or ICOSL (brown in c and pink in d) and CD3 (pink in b), CD11c (pink in c) and PDPN (brown in d). (e-f) Representative RNAscope microphotographs of human salivary glands of patients with Sjögren's syndrome with PDPN (red), CD11c (yellow) and ICOSLRNA (e) or DapBRNA (f) (green). (g) Graph showing absolute number and percentage of ICOSLRNA+ cells in pdpn+ immunofibroblasts and CD11c+ dendritic cells in with Sjögren's patient salivary gland.

Supplementary Fig. 5. Lymphocytes infiltrating salivary glands of Sjögren's Syndrome patients express LTα3. (a) Representative flow cytometry plots showing LTα3 producing CD3+ and CD19+ lymphocytes within mSGs of Sjögren's patients. Isotype control staining is also shown

Supplementary Fig. 6. LTα expression in serum is not a good predictor of local inflammation within salivary glands of Sjögren's patients. (a) Graph showing serum levels of LTα in sicca (clear squares) and Sjögren's Syndrome patients. (black squares). Data are representative as mean ± s.d., Mann-Whitney test. (b) Univariate analysis showing that serum LTα cannot be used as a predictor value for

Sjögren's Syndrome. (c) Spearman's correlation test shows lack of correlation between serum and saliva levels of LTα.

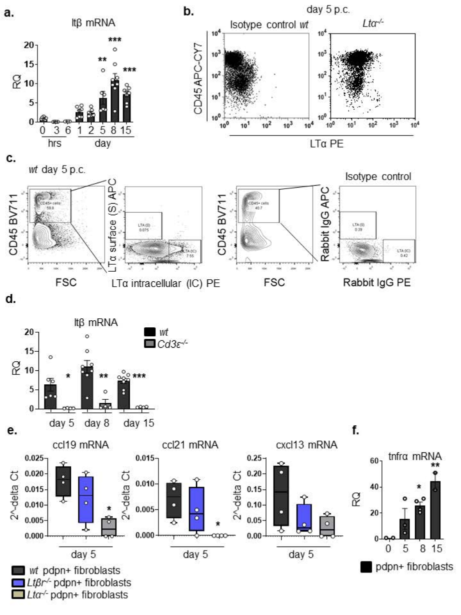
a.
ltβ mRNA
RQ
0 3 6
hrs
1 2 5 8 15
day
b.
day 5 p.c.
Isotype control wt
Ltα-/-
CD45 APC-CY7
LTα PE
c.
wt day 5 p.c.
CD45 BV711
FSC
LTα surface (S) APC
LTα intracellular (IC) PE
Isotype control
Rabbit IgG APC
Rabbit IgG PE
d.
ltβ mRNA
wt
Cd3ε-/-
day 5
day 8
day 15
e.
ccl19 mRNA
ccl21 mRNA
cxcl13 mRNA
2^-delta Ct
day 5
wt pdpn+ fibroblasts
Ltβr-/- pdpn+ fibroblasts
Ltα-/- pdpn+ fibroblasts
f.
tnfrα mRNA
pdpn+ fibroblasts

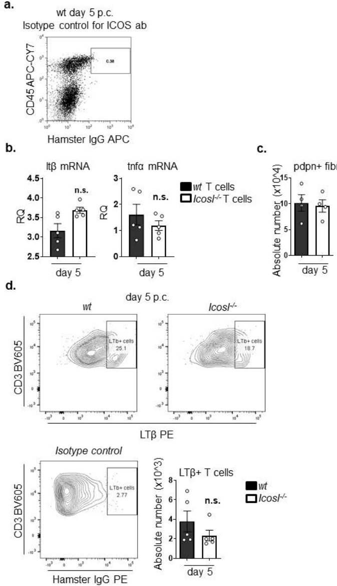
a.
wt day 5 p.c.
Isotype control for ICOS ab
CD45 APC-CY7
0.38
Hamster IgG APC
b.
ltβ mRNA
RQ
n.s.
day 5
tnfα mRNA
RQ
n.s.
day 5
wt T cells
Icosl-/- T cells
c.
pdpn+ fibroblasts
Absolute number (x10^4)
day 5
wt
Icosl-/-
d.
day 5 p.c.
wt
Icosl-/-
CD3 BV605
LTb+ cells
25.1
LTb+ cells
18.7
LTβ PE
Isotype control
CD3 BV605
LTb+ cells
2.77
Hamster IgG PE
LTβ+ T cells
Absolute number (x10^3)
n.s.
day 5
wt
Icosl-/-

a. **b.**

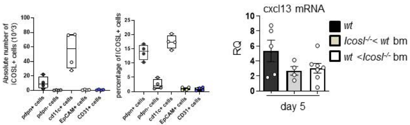
Absolute number of ICOSL+ cells (10^3)
100
80
60
40
20
0
pdpn+ cells
pdpn- cells
cd11c+ cells
EpCAM+ cells
CD31+ cells
percentage of ICOSL+ cells
25
20
15
10
5
0
cxcl13 mRNA
RQ
10
8
6
4
2
0
day 5
wt
Icosl-/-< wt bm
wt <Icosl-/- bm

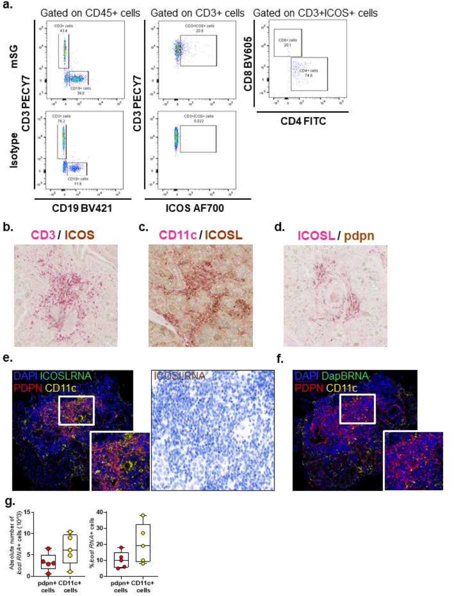
a.
Gated on CD45+ cells
Gated on CD3+ cells
Gated on CD3+ICOS+ cells
mSG
Isotype
CD3 PECY7
CD3 PECY7
CD8 BV605
CD4 FITC
CD19 BV421
ICOS AF700
b.
CD3 / ICOS
c.
CD11c / ICOSL
d.
ICOSL / pdpn
e.
DAPI ICOSLRNA
PDPN CD11c
ICOSLRNA
f.
DAPI DapBRNA
PDPN CD11c
g.
Absolute number of Icosl RNA+ cells (10^3)
% Icosl RNA+ cells
pdpn+ cells
CD11c+ cells
pdpn+ cells
CD11c+ cells

a.

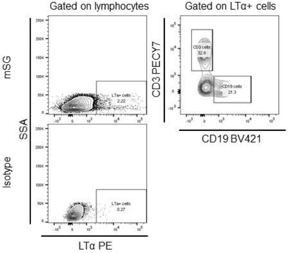

Gated on lymphocytes
Gated on LTα+ cells
mSG
Isotype
SSA
LTα+ cells
2.22
LTα+ cells
0.27
LTα PE
CD3 PECY7
CD3 cells
32.8
CD19 cells
21.3
CD19 BV421

a.

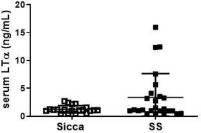

b.

	Unadjusted model		Adjusted model	
	Coeff. β (97,5% CI)	*p value*	*Coeff. β (95% CI)*	*p value*
Ltα	0.87 (0.67, 1.04)	0.20	-	-

Results of univariate analysis (unadjusted model) logistic regression of serum predictors of Sjögren syndrome.

c.

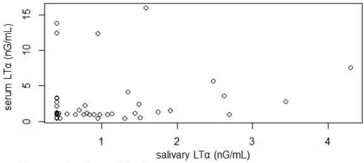

Spearman's rank correlation rho

Supplementary Table 1: Saliva proteins in Sjögren's Syndrome and Sicca patients

	SS patients	Sicca patients	*p*-value
hGDNF	1.01 (1.01 – 1.18)	1.01 (1.01 – 1.01)	0.030§
CDCP1	7.06 (6.47 - 7.74)	6.32 (5.90 - 6.71)	0.00016*
IL 6	6.06 (5.17 – 6.98)	5.43 (4.50 - 5.90)	0.033§
CXCL11	5.18 (2.57 - 6.12)	3.00 (1.77 - 4.56)	0.027§
CXCL9	9.07 (7.96 - 11.77)	8.20 (7.67 - 9.02)	0.025§
CCL4	3.82 (2.56 - 4.40)	2.22 (1.81 - 3.22)	0.023§
**CCL19**	**4.53 (2.40 – 5.90)**	**2.63 (1.47 - 3.39)**	**0.0025***
IL 15RA	0.65 (0.44 - 1.02)	0.39 (0.36 - 0.64)	0.0077§
IL 10RB	4.49 (4.23 - 5.23)	4.32 (4.16 - 4.87)	0.046*
PD-L1	2.32 (1.96 - 2.84)	2.03 (1.68 - 2.33)	0.0013§
RANKL	2.29 (1.40 - 3.29)	1.49 (0.96 - 1.75)	0.00015§
IL 12β	1.99 (0.82 - 3.98)	0.90 (0.55 - 1.42)	0.0069*
IL 13	0.75 (0.75 – 0.75)	0.75 (0.75 – 0.75)	0.049*
MMP-10	9.06 (7.05 - 10.49)	7.84 (7.21 - 8.43)	0.0010*
CD5	3.95 (3.31 – 4.70)	3.47 (3.11 - 3.82)	0.014§
MIP-1α	3.19 (2.13 - 4.08)	2.19 (1.30 - 3.96)	0.020§
Flt3L	3.74 (2.84 - 4.54)	3.16 (2.64 - 3.36)	0.016§
CXCL10	9.67 (7.61 - 12.60)	7.66 (6.18 - 9.80)	0.027§
CD40	9.18 (8.49 - 10.09)	8.86 (8.33 - 9.31)	0.032*
CCL20	6.84 (5.57 - 8.60)	4.99 (4.06 - 6.33)	0.0024§
**LTα**	**1.00 (0.42 - 2.09)**	**0.42 (0.40 - 0.78)**	**0.0013***

Continuous values are expressed as medians (25th–75th percentile)

§Wilcoxon rank-sum test

* Chi-square test

Supplementary Table 2: Univariate analysis (unadjusted model) logistic regression and multivariate analysis (adjusted model) of saliva predictors of Sjögren's syndrome.

	**Unadjusted model**		**Adjusted model**	
	Coeff. β (97,5% CI)	*p value*	*Coeff. β (95% CI)*	*p value*
**CCL19**	**-0.34 (-0.62, -0.11)**	**0.0076**	-	-
CXCL11	-0.24 (-0.48, -0.038)	0.0031	-	-
CXCL9	-0.39 (-0.72, -0.10)	0.012	-	-
CDCP1	-0.89 (-1.55, -0.32)	0.0042	-	-
IL15 RA	-2.20 (-4.20, -0.59)	0.015	-	-
PD-L1	-1.15 (-2.22, -0.36)	0.016	-	-
RANKL	-1.16 (-1.99, -0.50)	0.0020	-	-
IL12β	-0.72 (-1.30, -0.25)	0.0061	-	-
MMP-10	-0.42 (-0.79, -0.098)	0.016	-	-
CD5	-0.67 (-1.30, -0.15)	0.020	-	-
MIP-1α	-0.34 (-0.69, -0.039)	0.041	-	-
Flt3L	-0.72 (-1.35, -0.22)	0.011	-	-
CXCL10	-0.20 (-0.40, -0.030)	0.028	-	-
CD40	-0.58 (-1.18, -0.054)	0.041	-	-
CCL20	-0.30 (-0.56, -0.076)	0.014	-	-
**LTα**	**-1.56 (-2.77, -0.66)**	**0.0031**	**-3.93 (-7.87, -1.13)**	**0.020**

Supplementary Table 3: Mouse flow cytometry antibodies

Target	Fluorochrome	Clone	Supplier	Panel
CD45 (all isoforms) LCA, Ly-5	PERCPCy5.5/ PECy7/ APC-Cy7/BV711	30-F11	ebiosciences	surface
Podoplanin (gp38)	PE/PECY7	ebio8.1.1	ebiosciences	surface
CD31 (PECAM-1)	FITC/PECy7	390	ebiosciences	surface
CD326 (EpCAM)	APC/PECy7	G8.8	ebiosciences	surface
CD3e	PECy7	145-2C11	BD biosciences	surface
B220 (CD45R)	PB/APCCy7	RA3-6B2	BD biosciences	surface
CD4	efluor 450	RM4-5	ebiosciences	surface
CD8α	PE-TexasRed	53-6.7	BD biosciences	surface
CD19	APC	eBio1D3 (1D3)	ebiosciences	surface
NK1.1	FITC	PK136	ebiosciences	surface
CD11c	PE/PECy7	N418	ebiosciences	surface
ICOS	APC/AF700	15F9/C398.4A	Biolegend	surface
ICOSL	PE/Biotin	HK5.3	Biolegend	surface
IL13	PE/efluor450	eBio13A	ebiosciences	intracellular
IL22	AF647	Poly5164		intracellular
LTA	unconjugated	aa49-202	LSBio	intracellular
LTB	unconjugated	BBF6	Kind gift from Prof. Jeff Browning	surface
Donkey anti-rabbit	RPE	-	Southern Biotech	intracellular
Donkey anti-rabbit	APC	-	Life Technologies	surface
Goat anti-armenian hamster	PE		BD	surface

Supplementary Table 4: Human flow cytometry antibodies

Target	Fluorochrome	Clone	Supplier	Panel
CD15	BV510	W6D3	Biolegend	surface
CD14	V500	M5E2	BD Horrizon	surface
Zombie	Aqua		Biolegend	surface
CD03	FITC	HIT3A	Biolegend	surface
CD4	PE-dazzle594	RPA-T4	Biolegend	surface
CD8	PercP Cy5.5	RPA-T8	BD	surface
CD16	APC Cy7	3G8	Biolegend	surface
CD19	BV605	HIB19	Biolegend	surface
CD56	BV785	5.1 H11	BioLegend	surface
ICOS	PE Cy7	C398.4A	Biolegend	surface
Ltα (TNF-β)	PE	359-81-11	Biolegend	intracellular
Mouse (IgG1, k)	PE	P3.6.2.8.1	eBioscience	intracellular
